# Long‐Term Effects of Real‐Time Remotely Supervised High‐Intensity Interval Training in Adolescents With Obesity

**DOI:** 10.1002/osp4.70169

**Published:** 2026-07-17

**Authors:** Fábio de Freitas, Mariana R. Zago, Maria Ângela Antônio, Maria Ângela Bellomo Brandão, António Videira‐Silva

**Affiliations:** ^1^ Department of Pediatrics Unicamp School of Medical Sciences São Paulo Brazil; ^2^ WHO Collaborating Centre for Adolescent Medicine and Training Faculdade de Medicina Clínica Universitária de Pediatria Universidade de Lisboa Lisbon Portugal; ^3^ CIDEFES (Research Centre in Sport, Physical Education, Exercise and Health) Universidade Lusófona Lisbon Portugal; ^4^ CIFI2D (Centre for Research, Training, Innovation, and Intervention in Sport) Universidade do Porto Porto Portugal

**Keywords:** adolescent, high‐intensity interval training, home care services, obesity, physical activity

## Abstract

**Background:**

High‐intensity Interval Training (HIIT) has emerged as an effective strategy in the management of adolescents with obesity, including delivery through real‐time remote models. What remains unclear is whether these clinical benefits are sustained once direct professional supervision is discontinued. This study aimed to analyze the effects of a 6‐month, real‐time, remotely supervised HIIT program, integrated into a multi‐component clinical obesity treatment on post‐intervention and 12‐month follow‐up outcomes after sessions were discontinued. Furthermore, the association between motivation dimensions and program engagement was explored.

**Methods:**

A quasi‐experimental study involving 96 adolescents with obesity (BMI z‐score ≥ 2) was assigned to an Experimental Group (EG; *n* = 50) and a Control Group (CG; *n* = 46). The EG completed a remote‐supervised HIIT program (∼20 min, 4 × /week) alongside standard care, whereas the CG received only standard care. Engagement was categorized into three levels based on the duration of participation. EG participants were included in analyses if they attended at least 80% of the training sessions during the intervention period. Assessments were conducted at baseline, 6 months, and 12 months.

**Results:**

Group‐by‐time interaction showed reductions in the EG for BMI (*β* = 2.1 kg/m^2^, *p* < 0.001), BMI z‐score (*β* = 0.2, *p* = 0.002), and WC (*β* = 3.3 cm, *p* = 0.004) post‐intervention. A significant interaction was observed for water intake (*β* = 0.3 L/day, *p* = 0.042). Within EG, improvements were found in flexibility (*β* = 4.1 cm, *p* < 0.001) and muscular endurance (*β* = 3.2reps, *p* = 0.016). No significant changes occurred during the follow‐up period. Appearance motivation was negatively associated with engagement (*r*_p_ = −0.29, *p* = 0.049).

**Conclusion:**

The real‐time, remotely supervised HIIT program integrated into a multi‐component clinical obesity treatment was an effective approach to promote weight loss and improve physical fitness, with effects sustained for at least 12 months of follow‐up. Appearance‐driven goals may be associated with lower program engagement.

**Trial Registration:**

Brazilian Clinical Trials Registry (ReBEC): RBR‐35hyxxv

## 1 Introduction

Insufficient physical activity (PA) and high sedentary time are consistently associated with adverse metabolic profiles in adolescents with obesity [1]. Despite clear international guidelines for daily moderate‐to‐vigorous PA, most adolescents do not meet these targets [2]. Concurrent behaviors (such as prolonged screen time), high consumption of ultra‐processed foods, and sugar‐sweetened beverages further disrupt energy balance and increase the risk of weight gain [3].

Current clinical guidance supports multi‐component obesity treatment models that include nutritional counseling, behavior change strategies, psychological support, and structured PA [4, 5, 6]. Incorporating PA into weight‐management interventions has been linked to improvements in body composition, cardiorespiratory fitness, metabolic markers, and psychosocial outcomes in adolescents with obesity [7]. However, maintaining these benefits depends on sustained behavioral changes, which remains one of the greatest challenges in obesity management. Dropout rates in weight‐management programs are high, and engagement in PA tends to decline over time [8]. Motivation for starting and maintaining PA is a key factor for long‐run engagement [9].

Digital health (eHealth) strategies have rapidly expanded as a means to increase access and mitigate barriers (such as travel, scheduling, and costs) in weight‐management interventions [7, 10, 11]. Synchronous, videoconference‐based delivery can maintain real‐time interaction with professionals, support monitoring, and potentially improve treatment fidelity when integrated into existing clinical services [12, 13]. Building on this infrastructure, remotely supervised PA sessions serve as a practical extension of multi‐component programs for geographically dispersed populations or with limited resources [7, 14, 15].

In remote‐delivered formats, High‐Intensity Interval Training (HIIT) appears to be an attractive option for adolescents, especially given the common challenges of time, motivation, and fitness‐level variability [15]. The HIIT protocols can be performed in relatively short sessions, may require minimal equipment, and have demonstrated positive effects on cardiorespiratory fitness, cardiometabolic markers, and body composition in adolescents, including those with obesity [14]. Acute HIIT sessions have also been shown to impact post‐exercise energy expenditure (such as excess post‐exercise oxygen consumption) [16] and appetite regulation [17]. Although previous short‐term results were promising [18], a critical translational gap remains: the persistence of these benefits once direct supervision ends, particularly in a real‐time remote‐delivery model. Furthermore, the role of motivational dimensions in long‐term adherence and maintenance is underexplored. Addressing these critical gaps is essential for developing scalable, effective weight‐management interventions suitable for real‐world healthcare settings.

This study aimed to analyze the effects of a 6‐month, real‐time, remotely supervised high‐intensity interval training (HIIT) program integrated into a multi‐component clinical obesity treatment on post‐intervention and 12‐month follow‐up outcomes after sessions were discontinued. Furthermore, the association between motivation dimensions and program engagement was explored.

## 2 Methods

### 2.1 Study Design

The study protocol was registered at the Brazilian Clinical Trials Registry (ReBEC) under the identification number RBR‐35hyxxv. No protocol deviations occurred after trial registration. This pragmatic, prospective, quasi‐experimental controlled trial was conducted and reported according to the Consolidated Standards of Reporting Trials (CONSORT)‐Outcomes 2025 guidelines [19]. The completed CONSORT‐Outcomes checklist is available in the Supporting Information S1.

#### 2.1.1 Participants

Adolescents aged 12–17 years, with obesity (BMI z‐score ≥ 2 for sex and age, according to WHO standards) [20], were consecutively recruited from the Child and Adolescent Obesity Outpatient Clinic at the Clinical Hospital of UNICAMP (Brazil) between September 2021 and January 2024. All participants who agreed to participate, and their respective guardians signed a free and informed consent form.

Exclusion criteria included: (i) disabling orthopedic or neurological conditions, (ii) diagnosed psychiatric disorders, (iii) participation in other structured weight‐loss or PA programs (excluding school‐based Physical Education); or (iv) lack of access to an internet‐connected device with a camera. All candidates underwent medical evaluation to assess contraindications for participating in the program (such as orthopedic limitations, arrhythmias, or severe hypertension), and medical authorization was required for participation.

Adolescents were selected through consecutive sampling [21], with admission based on the order of arrival, in accordance with the outpatient clinic's routine. Those meeting the eligibility criteria were invited to participate. Due to the behavioral and remote nature of the intervention, randomization was considered operationally and behaviorally impractical in this real‐world outpatient context, as forced allocation could negatively affect adherence and increase attrition. Blinding was also not feasible due to the overt nature of the behavioral intervention and the direct interaction between participants and the professional during live sessions.

Thus, adolescents who agreed to participate in the HIIT program were assigned to the Experimental Group (EG), while those who declined the sessions but agreed to stay in the study and complete the assessments formed the Control Group (CG).

### 2.2 Timeline

The protocol was implemented in two stages, each lasting 6 months: Intervention and Follow‐up phases. The CG received standard outpatient care, including initial medical evaluation, nutritional counseling, and follow‐up appointments as per the Brazilian Society of Pediatrics guidelines for pediatric obesity [22] along with national dietary [23] and PA recommendations [24].

In addition to standard care, the EG participated in a real‐time, remotely supervised HIIT program conducted via videoconference by the same certified physical education specialist experienced in pediatric obesity treatment. Sessions were held four times per week (∼20 min each) for 6 months. Each session comprised: 5 min of dynamic warm‐up; ∼10 min of circuit‐based interval training (aerobic and resistance bodyweight exercises, e.g., squats, mountain climbers, planks), structured in ∼4 sets of 40 s work/10 s rest, and 5 min of cool‐down. Training intensity and volume were progressively adapted to individual fitness levels with continuous monitoring of heart rate and perceived exertion. Detailed descriptions of the training protocol were previously published [18]. In the follow‐up, the remote‐supervised HIIT sessions were discontinued, and EG participants received only standard care during the 6‐ to 12‐month follow‐up. Throughout the entire 12‐month study, participants in both groups attended outpatient appointments at baseline, 3, 6, and 9 months, following the standard clinical routine. After starting the protocol, it continued as originally planned with no significant adjustments made.

### 2.3 Program Engagement

Participants were categorized into three groups based on engagement level (i.e., months of participation in the study): (i) Low engagement: Participants who did not complete the 6‐month intervention phase (e.g., non‐adherence, dropout, or engagement for ≤ 3 months); (ii) Moderate engagement: Participants who completed the 6‐month intervention phase but did not complete the subsequent 6‐month follow‐up (i.e., 6 months total engagement); and (iii) High engagement: Participants who completed the entire 12‐month study period (Intervention and Follow‐up phases).

The EG participants were considered eligible for analysis if they attended at least 80% of the training sessions during the intervention phase.

### 2.4 Outcomes

Primary outcomes included 6‐ and 12‐month changes in anthropometrics and body composition (Waist Circumference, BMI, BMI z‐score, body fat mass percentage, and skeletal muscle mass percentage [SMM]) and in physical fitness (flexibility and muscular endurance).

Secondary outcomes included: sleep duration, nutritional habits (water intake, sugary drink consumption, and total calorie intake), and engagement. All the secondary outcomes were analyzed at the same time points (baseline, 6, and 12 months).

### 2.5 Measures

Information on comorbidities was retrieved from the participants' electronic medical records and recorded to identify the most prevalent conditions.

Anthropometric (weight, Height, waist circumference), body composition (BMI, BMI z‐score, body fat mass percentage, and skeletal muscle mass percentage), and physical fitness (flexibility and muscular endurance) assessments were performed according to standardized procedures, as previously published [18].

Nutritional information was assessed with 24‐h dietary recalls through structured interviews conducted by trained staff, as previously detailed [25].

Motivational dimensions for PA were assessed using the Portuguese version of the Motives for Physical Activity Measure‐Revised (MPAM‐R) as previously detailed [26].

### 2.6 Statistical Analyses

Data normality was assessed using the Shapiro–Wilk test and visual inspection of the histogram. Continuous variables were reported as mean ± standard deviation for normally distributed data and as median [interquartile range, IQR] for non‐normally distributed data. Baseline differences between EG and CG were tested with independent‐sample *t*‐tests or Mann–Whitney *U* tests for normally distributed and non‐normally distributed data, respectively, and Pearson's chi‐square test for categorical variables.

For longitudinal analyses (baseline to 6 months, and 6–12 months), linear quantile mixed‐effects regression models (lqmm, R) were run to address non‐normal distributions. Fixed effects included group (EG vs. CG), time (baseline vs. 6 months), and their interaction, with the EG as the reference category, to interpret the HIIT effect. Follow‐up analysis (6–12 months) included completers only.

Motivational scores were compared across engagement groups using Kruskal–Wallis tests with Dunn's post hoc contrasts. Partial Spearman correlations, adjusted for age and sex, were used to analyze the relationships between motivational dimensions and program engagement.

Analyses were conducted using IBM SPSS Statistics v28.0 (IBM Corp., Armonk, NY, USA) and R v4.3.1 (R Foundation for Statistical Computing). All tests were two‐tailed, with significance set at *p* < 0.05.

## 3 Results

### 3.1 Baseline Characteristics

**FIGURE 1:**
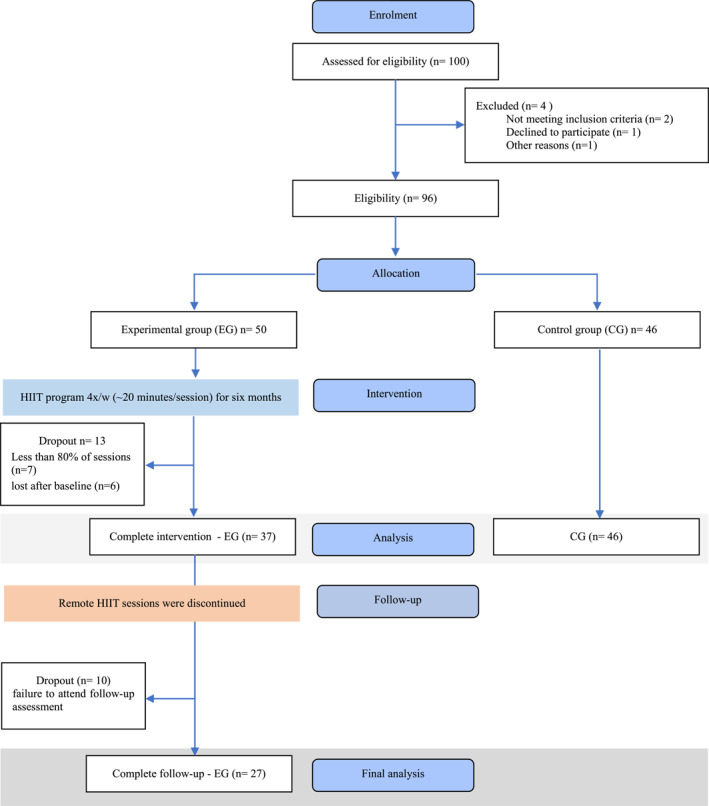
Participant flow diagram of the remote‐supervised HIIT program trial. Flow diagram illustrating participant progress through the phases of the quasi‐experimental controlled trial: enrollment, group allocation, intervention delivery, follow‐up, and analysis.

Figure 1 illustrates the flow of participants throughout the study, including allocation, adherence, and follow‐up. Of the 100 adolescents assessed, 96 met the inclusion and exclusion criteria. These participants were then assigned to the EG (*n* = 50) or the CG (*n* = 46).

Despite the pragmatic allocation, the EG exhibited significantly higher body weight (*U* = 799.5, *p* = 0.010), BMI (*U* = 754.5, *p* = 0.004), BMI z‐score (*U* = 700.5, *p* = 0.001), and WC (*U* = 788.0, *p* = 0.008), than the CG at baseline. Additionally, the EG reported significantly lower sugary beverage consumption (*U* = 588.5, *p* < 0.001) and a lower prevalence of asthma (*χ* ^2^ = 4.03, *p* = 0.045). No other significant differences were observed between the groups at baseline (Table 1).

**TABLE 1:** Baseline characteristics of participants.

Variables	Control	Experimental	*p‐value*
Age (years)	13.8 [1.7]	13.8 [2.4]	0.944^b^
Sex, *n* (%)			
Male	26 (56,5)	30 (60.0)	0.730^c^
Female	20 (43.5)	20 (40.0)	
Anthropometry and body composition			
Weight (kg)	86.0 [25.8]	103.5 [40.4]	**0.010** ^b^
Height (cm)	164.4 ± 9.0	165.7 ± 9.2	0.439^a^
WC (cm)	95.0 [16.8]	102.5 [20.3]	**0.008** ^b^
BMI (kg/m^2^)	31.6 [8.5]	36.5 [11.5]	**0**.**004** ^b^
BMI z‐score	2.84 [1.02]	3.41 [1.53]	**0**.**001** ^b^
BFM (%)	37.4 ± 4.8	36.8 ± 6.4	0.712^a^
Movement behavior			
Screen time (h/day)	4.0 [3.0]	5.0 [3.0]	0.459^b^
Sleep duration (h/day)	8.0 [2.0]	8.0 [2.0]	0.676^b^
Nutritional behaviors			
Water intake (L/day)	1.0 [1.0]	1.0 [1.0]	0.937^b^
Sugary beverages (mL/day)	300.0 [213.0]	200.0 [300.0]	**< 0.001** ^b^
Calories (kcal/day)	1729.4 ± 564.7	1619.2 ± 549.1	0.301^a^
Comorbidities *n* (%)			
SAH	13 (28.3)	17 (34.0)	0.544^c^
Insulin resistance	7 (15.2)	10 (20.0)	0.540^c^
Anxiety	6 (13.0)	6 (12.0)	0.877^c^
Hepatic steatosis	5 (10.9)	4 (8.0)	0.630^c^
Asthma	9 (19.6)	3 (6.0)	**0.045** ^c^
≥ 1 comorbidity	7 (15.2)	11 (22.0)	0.395^c^

*Note:* Mean ± SD: standard deviation; Median [Interquartile range]; *n* (%): sample (percentage); Bold values indicate statistically significant differences (*p* ≤ 0.05).

Abbreviations: BFM: body fat mass; BMI: body mass index; SAH: Systemic arterial hypertension; WC: waist circumference.

^a^ Independent samples t‐tests for normally distributed variables.

^b^ Mann–Whitney *U* tests for non‐normally distributed variables.

^c^ Pearson's chi‐square test for categorical variables.

No statistically significant differences were observed at baseline across the engagement groups (Table S1).

### 3.2 Effects of Intervention

Of the 50 participants allocated to the EG, 13 (26.0%) did not complete the 6‐month assessment due to non‐adherence (*n* = 7) or dropout (*n* = 6), resulting in 37 participants included in post‐intervention analyses. No adverse events were reported during or as a consequence of the remote‐supervised HIIT program.

Group‐by‐time interaction showed greater reductions in the EG for WC (*β* = 3.3 cm, 95% CI: 1.1 to 5.5, *p* = 0.004), BMI (*β* = 2.1 kg/m^2^, 95% CI: 1.0 to 3.1, *p* < 0.001), and BMI z‐score (*β* = 0.2, 95% CI: 0.1 to 0.4, *p* = 0.002) compared to CG. A smaller but significant interaction was also observed for water intake (*β* = 0.3 L/day, 95% CI: 0.0 to 0.6, *p* = 0.042) (Table 2).

**TABLE 2:** Changes in primary and secondary outcomes from baseline to 6 months.

Variables	Intervention							
	Control		Experimental		Time^a^		Group*Time^b^	
Anthropometry and body composition	Baseline	Month 6	Baseline	Month 6	*β* (95% CI)	*p‐value*	*β* (95% CI)	*p‐value*
WC (cm)	95.0 [16.8]	96.0[17.5]	102.5 [20.3]	101.0 [17.5]	−2.7 (−4.5, −0.8)	**0.006**	3.3 (1.1, 5.5)	**0.004**
BMI (kg/m^2^)	31.6 [8.5]	33.6 [10.0]	36.5 [11.5]	35.6 [10.3]	−1.2 (−2.2, −0.2)	**0.016**	2.1 (1.0, 3.1)	**< 0.001**
BMI z‐score	2.84 [1.02]	2.92 [1.10]	3.41 [1.53]	3.28 [1.26]	−0.3 (−0.4, −0.1)	**< 0.001**	0.2 (0.1, 0.4)	**0.002**
BFM (%)	37.4 ± 4.8	36.9 ± 5.7	36.8 ± 6.4	35.3 ± 6.6	−1.1 (−2.7, 0.5)	0.162	1.1 (−0.5, 2.7)	0.173
SMM (%)	—	—	62.1 [8.6]	63.4 [10.5]	1.2 (0.1, 2.4)*	**0.040**	—	—
Physical fitness								
Flexibility (cm)	—	—	22.5 [14.0]	28.0 [14.5]	4.1 (2.4, 5.8)*	**< 0.001**	—	—
Muscular endurance (sit‐ups)	—	—	16.0 [16.3]	20.0 [15.0]	3.2 (0.6, 5.8)*	**0.016**	—	—
Movement behavior								
Screen time (h/day)	4.0 [3.0]	4.0 [3.0]	5.0 [3.0]	4.0 [3.0]	−0.2 (−1.2, 0.8)	0.663	0.3 (−0.7, 1.4)	0.545
Sleep duration (h/day)	8.0 [2.0]	8.0 [2.0]	8.0 [2.0]	8.0 [2.0]	−0.2 (−0.5, 0.2)	0.394	−0.1 (−0.7, 0.5)	0.716
Nutritional behaviors								
Water intake (L/day)	1.0 [1.0]	2.0 [1.5]	1.0 [1.0]	2.0 [1.0]	0.2 (0.0, 0.4)	0.094	0.3 (0.0, 0.6)	**0.042**
Sugary beverages (mL/day)	300.0 [213.0]	283.0 [132.5]	200.0 [300.0]	100.0 [200.0]	−23,8 (−98.1, 50.6)	0.524	−36.5 (−136.6, 63.6)	0.467
Calories (kcal/day)	1729.4 ± 564.7	1733.3 ± 532.1	1619.2 ± 549.1	1571.0 ± 536.0	−52.1 (−256.2, 152.1)	0.611	44.9 (−233.5, 323.3)	0.747

*Note:* Mean ± SD: standard deviation; Median [Interquartile range]; Regression analysis presented *β* (95% CI); Bold *β* indicates a significant interaction (*p* ≤ 0.05).

Abbreviations: BFM: body fat mass; BMI: body mass index; CI: confidence interval; SMM: skeletal muscle mass; WC: waist circumference.

^a^ β Time represents the median change within the EG with reference category; (*) analysis was performed only within the EG due to the absence of comparable data for the CG; (−) data were not collected for the respective group or outcome.

^b^ β Group*Time represents the differential median change in CG relative to EG (EG: reference category); Models are adjusted for sex and age.

Within EG, significant improvements were observed with a reduction in WC (*β* = −2.7 cm, 95% CI: −4.5 to −0.8, *p* = 0.006), BMI (*β* = −1.2 kg/m^2^, 95% CI: −2.2 to −0.2, *p* = 0.016), and BMI z‐score (*β* = −0.3, 95% CI: −0.4 to −0.1, *p* < 0.001).

An increase was observed in flexibility (*β* = 4.1 cm, 95% CI: 2.4 to 5.8, *p* < 0.001) and muscular endurance (*β* = 3.2 reps, 95% CI: 0.6 to 5.8, *p* = 0.016). No significant changes were observed in movement or nutritional behaviors (Table 2).

### 3.3 Effects in the Follow‐Up

Among the 37 EG participants included in the 6‐month analysis, 27 (73.0%) completed the 12‐month follow‐up. Within this group, linear quantile mixed‐model regression revealed no statistically significant changes in body composition, physical fitness, movement behavior, or nutritional behaviors during the 6–12‐month follow‐up period (Table 3).

**TABLE 3:** Longitudinal changes in primary and secondary outcomes within the experimental group (6–12 months follow‐up; *n* = 27).

Variables	Follow‐up			
	month 6	month 12	Time^a^	*p‐value*
Anthropometry and body composition				
WC (cm)	98.8 ± 10.4	97.3 ± 10.5	−1.3 (−3.1, 0.5)	0.144
BMI (kg/m^2^)	35.1 ± 4.8	35.1 ± 5.3	−0.2 (−1.0, 0.7)	0.725
BMI z‐score	3.20 [1.02]	3.03 [0.68]	−0.07 (−0.21, 0.07)	0.307
BFM (%)	35.1 ± 6.1	34.8 ± 6.1	−0.3 (−1.7, 1.1)	0.684
SMM (%)	64.9 ± 6.2	65.3 ± 6.3	0.4 (−0.9, 1.6)	0.588
Physical fitness				
Flexibility (cm)	28.0 [14.5]	26.0 [13.5]	−0.7 (−2.3, 0.9)	0.364
Muscular endurance (sit‐ups)	20.0 [15.0]	22.0 [16.8]	0.7 (−1.1, 2.5)	0.434
Movement behavior				
Screen time (h/day)	4.0 [3.0]	5.0 [5.0]	0.4 (−0.4, 1.2)	0.351
Sleep duration (h/day)	8.0 [2.0]	8.0 [2.0]	−0.03 (−0.6, 0.5)	0.924
Nutritional behaviors				
Water intake (L/day)	2.0 [1.0]	1.5 [1.0]	0.1 (−0.3, 0.4)	0.634
Sugary beverages (mL/day)	100.0 [200.0]	0.0 [280]	0.4 (−45.0, 45.9)	0.984
Calories (kcal/day)	1530.0 [925.0]	1395.0 [700.0]	−66.2 (−308.1, 175.8)	0.585

*Note:* Data presented as Mean ± SD or Median [Interquartile Range]. Analyses are restricted to participants who completed the 12‐month follow‐up of the EG (*n* = 27). *β* represents the median change from 6 to 12 months.

Abbreviations: BFM: body fat mass; BMI: body mass index; CI: confidence interval; SMM: skeletal muscle mass; WC: waist circumference.

^a^ Linear quantile mixed‐effects regression models (lqmm) adjusted for age and sex, with participant ID as a random intercept; *β* (95% CI).

### 3.4 Motivational Dimensions and Engagement

At baseline, motivational profiles were largely similar across engagement categories, except for appearance‐related motives, which showed a negative association with program engagement (*r*_p_ = −0.29, *p* = 0.049). No statistically significant differences in motivational scores were observed among engagement categories (Table 4).

**TABLE 4:** Baseline motivational dimensions across program engagement categories and their association.

	Group comparison				Partial spearman correlation		Quantile regression	
Motives	Low	Moderate	High	*p‐value* ^a^	*r*_*p*_	*p‐value* ^b^	*β* (95% CI)	*p‐value* ^c^
Enjoyment	6.0 [1.9]	4.1 [3.1]	4.7 [2.2]	0.090	−0.20	0.170	−0.4 (−2.4, 1.7)	0.729
Competence	5.9 [1.9]	5.2 [3.7]	4.6 [3.9]	0.188	−0.26	0.080	−0.8 (−1.8, 0.3)	0.166
Appearance	6.5 [1.1]	5.6 [2.2]	5.7 [2.4]	0.089	−0.29	**0.049**	−1.2 (−2.6, 0.1)	0.071
Fitness	7.0 [1.3]	7.0 [1.1]	6.7 [0.8]	0.784	0.00	1.000	1.2 (−1.0, 3.4)	0.280
Social	4.7 [2.6]	3.5 [2.7]	3.4 [3.1]	0.237	−0.16	0.277	0.6 (−1.3, 2.5)	0.534

*Note:* Mean ± SD: standard deviation; Median [Interquartile range]. Motives: MPAM‐R: Motives for Physical Activity Measure–Revised.

^a^ Kruskal–Wallis tests were used to compare motivational scores across Program engagement categories.

^b^ Partial Spearman correlation coefficients (*r*_p_) were calculated and adjusted for age and sex to examine relationships with Program engagement. Bold correlation coefficients indicate statistical significance (*p* < 0.05).

^c^ Linear quantile mixed‐effects regression models (lqmm), adjusted for age and sex, with participant ID as a random intercept; *β* (95% CI).

## 4 Discussion

The remotely supervised HIIT program, integrated with a multicomponent clinical treatment for obesity in adolescents, was effective in promoting weight loss and improving body composition and physical fitness, with sustained effects for at least 12 months of follow‐up. Adolescents driven primarily by appearance‐related goals were less likely to sustain participation, whereas those with more self‐determined motives showed greater adherence.

The remotely supervised HIIT program integrated with a multicomponent treatment led to significant improvements in body composition compared with standard treatment alone. The group‐time interaction analyses showed reductions in BMI, BMI z‐score, and WC in the EG compared with the CG. The results obtained reinforce previous studies' observations, which highlight the effectiveness of HIIT in reducing excess weight in adolescents with obesity [27]. For example, Meng et al. [14] observed significant reductions in BMI after an HIIT‐based intervention performed in a school setting. The central point of our contribution to the management of obesity in the outpatient clinic goes far beyond weight reduction. The effect achieved in face‐to‐face interventions can also be achieved through a remote supervision model. These findings support the remote approach as a viable alternative with the potential to expand access to treatment.

The significant increase in SMM observed in EG after 6 months is in line with previous research, which points to the potential of HIIT‐based interventions to stimulate gains in SMM percentage [28]. As a key component of fat‐free mass, SMM is metabolically active and contributes 60%–70% of total daily energy expenditure, reinforcing its strategic role in the management of obesity in adolescents [29]. Although comparable data for the CG on this outcome were not available, the pattern of improvement observed in the EG suggests that remotely supervised HIIT may be a promising alternative to promote muscle gains in this population.

In terms of physical fitness, the EG showed significant improvements, including approximately 24% increase in flexibility and 25% increase in muscular endurance. The absence of corresponding CG data limits direct causal inference, but the magnitude of the gains and the consistency with previously reported evidence strengthen the interpretation that the intervention played a relevant role in these outcomes [30]. Despite the need for caution when interpreting these direct‐comparator results, the observed improvement pattern suggests the potential of the remotely supervised HIIT program to enhance physical fitness.

A secondary finding was a significant increase in water consumption in the EG, especially over the intervention period, when the observed increase was 28.6%. This pattern aligns with the literature, which indicates that regular structured PA can promote more appropriate hydration behaviors [31]. It is possible that this change reflects a greater perception of hydration needs during sessions, a perception that tends to consolidate with session repetition. Although it is a less central outcome, it highlights the educational reach of supervised interventions, which can influence daily health behaviors [32].

The maintenance of gains in body composition and physical fitness over the 12‐month follow‐up period emerges as a clinically relevant result. The absence of regression after discontinuation of direct remote supervision is a positive indicator, something that, in long‐term behavioral interventions, is not always sustained. This behavior can be interpreted in the light of the Self‐Determination Theory (SDT), which emphasizes the role of autonomous motivation in maintaining health practices [33]. However, this internalization did not occur uniformly. By offering an initial period of supervised practice, even if remote, the program may have facilitated and helped internalize the value of the PA, an essential step for the behavior to be maintained even without external requests [34]. In addition, standard outpatient follow‐up, including periodic visits and ongoing health monitoring, likely worked as an additional, discreet but important support, helping to reduce the risk of relapse [35].

The results revealed a clear behavioral pattern: the motivational fitness dimension was the primary driver of initial program involvement. On the other hand, motivation related to appearance was negatively associated with program involvement. This is consistent with evidence that adolescents guided by extrinsic goals, especially those linked to body image, tend to create unrealistic expectations that, when not achieved, result in frustration and abandonment [36].

Unlike systematic reviews and meta‐analyses in which intrinsic motivation appears as the main driver of PA practice, the clinical context of this study seems to have shaped a more fitness‐centered pattern. This is possibly due to the nature of the messages to which adolescents are exposed, usually focused on health, prevention, and improvement of physical performance [9]. However, intrinsic motives based on pleasure and perceived competence may be associated with greater persistence in behavior [33]. In this study, these two dimensions showed an inverse correlation with weight, suggesting that as adolescents felt more pleasurable and capable, weight tended to decrease.

## 5 Limitations

This study has limitations that may affect the generalizability of results. The small sample size, combined with follow‐up dropouts, reduced the statistical power of some secondary analyses. A dropout rate of 26% was observed during the intervention phase, with an additional 27% loss during follow‐up. This attrition may have been influenced by the program's length, the characteristics of the population served, and the real‐world clinical context, which traditionally poses challenges to prolonged engagement.

The sample reflected the natural flow of patients seen in a clinical setting, where adherence difficulties are commonly observed among adolescents with obesity [8]. Family‐related factors, unstable home environments, or limited parental involvement may have contributed to participant attrition. Although parental involvement is recognized as an important factor in obesity interventions [37], formal data on parental participation were not collected. In addition, socioeconomic characteristics were not assessed, which limits the interpretation of potential barriers related to internet access, remote participation, and treatment engagement. Nevertheless, all participants were referred by the public health system, which may have resulted in some degree of socioeconomic homogeneity within the sample. Furthermore, behavioral outcomes were self‐reported and may therefore be subject to recall bias or social desirability bias.

A significant limitation in the reporting of physical fitness and body composition outcomes was the unavailability of comparable baseline and follow‐up data for SMM, flexibility, and muscular endurance in the CG. This gap undermines causal inference about the gains observed in the EG. Although intragroup analyses indicate significant improvements in these variables, such findings should be interpreted with caution.

Although PA is widely recognized as a core component of behavior change and obesity management in adolescents [38], integrating structured PA interventions into routine clinical services remains difficult [39]. Clinical guidelines highlight the importance of PA in treating obesity [40]; however, much of the existing evidence comes from controlled research environments that do not fully reflect the operational constraints, adherence differences, and logistical challenges present in public health systems. The current results help fill this gap by demonstrating that a real‐time, remotely supervised HIIT protocol can be effectively used in a public outpatient obesity service. It's important to note that this intervention was carried out under routine clinical conditions and incorporated into a multicomponent treatment model, reflecting the complexity and variation typical of real‐world care. The ongoing benefits beyond the supervised phase further highlight the potential long‐term effectiveness of this practical, service‐centered approach [41], supporting the integration of structured, remotely supervised PA programs as a feasible and sustainable option within standard outpatient obesity management.

## 6 Conclusion

This study found that the real‐time, remotely supervised HIIT program integrated into a multicomponent clinical treatment for obesity in adolescents was an effective approach to promote weight loss and improve physical fitness, with sustained effects for at least 12 months of follow‐up. Adolescents driven primarily by appearance‐related goals were less likely to sustain participation, whereas those with more self‐determined motives showed greater adherence.

The absence of adverse events, combined with consistent results, supports the feasibility and safety of implementing this model within routine clinical services. Such programs represent a viable and scalable alternative for adolescents with limited access to face‐to‐face services, contributing significantly to public health strategies for obesity management.

## Author Contributions

**Fábio de Freitas:** conceptualization, data curation, formal analysis, investigation, methodology, writing – original draft, writing – review and editing. **Mariana R. Zago:** investigation, visualization, writing – review and editing. **Maria Ângela Antônio:** visualization, supervision, writing – review and editing. **Maria Ângela Bellomo Brandão:** funding acquisition, project administration, resources, supervision, validation, visualization, writing – review and editing. **António Videira‐Silva:** conceptualization, data curation, supervision, validation, writing – review and editing.

## Funding

This study was financed in part by the Coordination for the Improvement of Higher Education Personnel (CAPES), Brazil—Finance Code 001, Grant No. 88887.637569/2021‐00.

## Ethics Statement

This study was approved by the Research Ethics Committee of the State University of Campinas under opinion number 4.940.045, following the norms established by Resolution 466/2012 of the National Health Council. All participants and their guardians who agreed to participate signed a free and informed consent form.

## Conflicts of Interest

The authors declare no conflicts of interest.

## Supporting information


Supporting Information S1

**Table S1:** Baseline characteristics by program retention category.

## Data Availability

Research data are not shared.
