# Supplementary material for: Long‐Term Effects of Real‐Time Remotely Supervised High‐Intensity Interval Training in Adolescents With Obesity
Source: Obes Sci Pract. 2026 Jul 17;12(4):e70169. doi: 10.1002/osp4.70169 (PMC13378902; doi:10.1002/osp4.70169)
Supplement: Supplementary file 2 — Table S1: Baseline characteristics by program retention category. [file OSP4-12-e70169-s002.docx]

**Supplemental Table.** Baseline characteristics by program retention category.

| **Variables** | **Low (n=13)** | **Moderate (n=10)** | **High (n=27)** | ***p-value*** |
| --- | --- | --- | --- | --- |
| Age (years) | 13.9 [3.7] | 14.8 [3.9] | 13.5 [1.7] | .549 ^b^ |
| **Sex, n (%)** |  |  |  | .768 ^c^ |
| Male | 8 (26.7) | 5 (16.7) | 17 (56.7) |  |
| Female | 5 (25.0) | 5 (25.0) | 10 (50.0) |  |
| **Anthropometry and body composition** |  |  |  |  |
| Weight (kg) | 102.1 ± 29.5 | 120.7 ± 36.4 | 98.9 ± 23.4 | .107 ^b^ |
| Height (cm) | 166.3 ± 11.5 | 168.5 ± 8.4 | 164.5 ± 8.5 | .477 ^a^ |
| WC (cm) | 102.2 ± 18.2 | 109.7 ± 15.5 | 101.0 ± 11.5 | .237 ^a^ |
| BMI (kg/m2) | 36.6 ± 8.0 | 42.4 ± 12.4 | 36.1 ± 5.9 | .103 ^a^ |
| BMI z-score | 3.04 [1.57] | 3.94 [2.26] | 3.40 [0.94] | .422 ^b^ |
| BFM (%) | 37.5 ± 5.8 | 38.1 ± 7.7 | 36.1 ± 6.3 | .586 ^a^ |
| SMM (%) | 62.1 ± 5.4 | 61.9 ± 7.7 | 63.9 ± 6.4 | .126 ^a^ |
| **Physical fitness** |  |  |  |  |
| Flexibility (cm) | 25.5 ± 10.1 | 20.7 ± 8.9 | 22.2 ± 8.2 | .597 ^a^ |
| Muscular endurance (sit-ups) | 13.7 ± 11.1 | 14.8 ± 9.8 | 14.9 ± 9.6 | .752 ^a^ |
| **Movement behavior** |  |  |  |  |
| Screen time (h/day) | 5.2 ± 2.4 | 4.9 ± 2.2 | 5.1 ± 2.9 | .962 ^a^ |
| Sleep duration (h/day) | 8.0 [2.3] | 8.0 [3.0] | 8.0 [1.0] | .987 ^b^ |
| **Nutritional behaviors** |  |  |  |  |
| Water intake (L/day) | 1.0 [0.3] | 2.0 [1.3] | 1.0 [1.0] | .147 ^b^ |
| Sugary beverages (ml/day) | 165.0 [339.0] | 250.0 [200] | 100 [200] | .646 ^b^ |
| Calories (kcal/day) | 1581.4 ± 512.9 | 1721.5 ± 655.8 | 1578.8 ± 513.1 | .770 ^a^ |
| **Comorbidities n (%)** |  |  |  |  |
| SAH | 2 (11.8) | 4 (23.5) | 11 (64.7) | .257 ^c^ |
| Insulin Resistance | 1 (10.0) | 4 (40.0) | 5 (50.0) | .161 ^c^ |
| Anxiety | 2 (33.3) | - | 4 (66.7) | .237 ^c^ |
| Hepatic Steatosis | - | 1 (25.0) | 3 (75.0) | .281^c^ |
| Asthma | 1 (33.3) | 1 (33.3) | 1 (33.3) | .745 ^c^ |
| ≥ 1 comorbidity | 2 (18.2) | 3 (27.3) | 6 (54.5) | .702 ^c^ |

Mean ± SD: standard deviation; n (%): sample (percentage); Median [Interquartile range];

1. One-Way ANOVA;
2. Kruskal-Wallis H Test.
3. Likelihood Ratio Chi-square test due to low expected cell counts.

WC: waist circumference; BMI: body mass index; BFM: body fat mass; SMM: skeletal muscle mass.
